# Case Report: Expanding the Genetic and Phenotypic Spectrum of Autosomal Recessive Spastic Ataxia of Charlevoix-Saguenay

**DOI:** 10.3389/fgene.2020.585136

**Published:** 2020-12-22

**Authors:** Parham Habibzadeh, Zahra Tabatabaei, Soroor Inaloo, Muhammad Mahdi Nashatizadeh, Matthis Synofzik, Vahid Reza Ostovan, Mohammad Ali Faghihi

**Affiliations:** ^1^Persian BayanGene Research and Training Center, Shiraz University of Medical Sciences, Shiraz, Iran; ^2^ Neonatal Research Center, Shiraz University of Medical Sciences, Shiraz, Iran; ^3^Parkinson’s Disease and Movement Disorder Center, Department of Neurology, University of Kansas School of Medicine, Kansas, KS, United States; ^4^ Department of Neurodegenerative Diseases, Hertie-Institute for Clinical Brain Research and Center of Neurology, University of Tübingen, Tübingen, Germany; ^5^German Center for Neurodegenerative Diseases (DZNE), Tübingen, Germany; ^6^Clinical Neurology Research Center, Shiraz University of Medical Sciences, Shiraz, Iran; ^7^Center for Therapeutic Innovation, Department of Psychiatry and Behavioral Sciences, University of Miami Miller School of Medicine, Miami, FL, United States

**Keywords:** ARSACS, rigidity, hypokinesia, spastic ataxia, early-onset ataxia, recessive ataxia

## Abstract

Autosomal recessive spastic ataxia of Charlevoix–Saguenay (ARSACS) is a rare neurodegenerative disorder caused by biallelic mutations in the *SACS* gene. Once thought to be limited to Charlevoix–Saguenay region of Quebec, recent evidence has indicated that this disorder is present worldwide. It is classically characterized by the triad of ataxia, pyramidal involvement, and axonal-demyelinating sensorimotor neuropathy. However, diverse clinical features have been reported to be associated with this disorder. In this report, we present the first Iranian family affected by ARSACS with unique clinical features (mirror movements, hypokinesia/bradykinesia, and rigidity) harboring a novel deletion mutation in the *SACS* gene. Our findings expand the genetic and phenotypic spectrum of this disorder.

## Background

First reported in the late 1970s, autosomal recessive spastic ataxia of Charlevoix–Saguenay (ARSACS; MIM#270550) is a neurodegenerative disorder characterized by early-onset spastic ataxia and sensorimotor neuropathy (Bouchard et al., 1978; Pilliod et al., 2015). Although initially reported in a series of patients in Quebec, numerous reports from other world regions has made this condition one of the most frequent causes of spastic ataxia worldwide (Vermeer et al., 1993; Synofzik et al., 2013; Synofzik and Nemeth, 2018; Kuchay et al., 2019). ARSACS is caused by pathogenic mutations in *SACS* gene (OMIM #604490), coding for a 520-kDa multidomain protein called sacsin, which is thought to integrate the 70-kDa heat shock protein (Hsp70) chaperone and ubiquitin–proteasome systems. Sacsin is most highly expressed in large neurons involved in the motor system, especially cerebellar Purkinje cells, where it has normally been found to protect against at least one mutation of the ataxin-1 gene that results in spinocerebellar ataxia type 1 (Parfitt et al., 2009). Recent evidence indicates that *SACS* domains regulate intermediate filament assembly (Gentil et al., 2019). *SACS*-knockout mice have also been shown to exhibit early ataxia and abnormal bundling of neurofilaments in different neuronal populations (Lariviere et al., 2015).

Advances in various genetic sequencing technologies have enabled us to expand the clinical and molecular spectrum of various hereditary ataxia disorders (Habibzadeh et al., 2019). Herein, we report on a novel homozygous *SACS* mutation in an Iranian family with ARSACS with unique clinical findings.

## Case Presentation

### Clinical Findings

**FIGURE 1:**
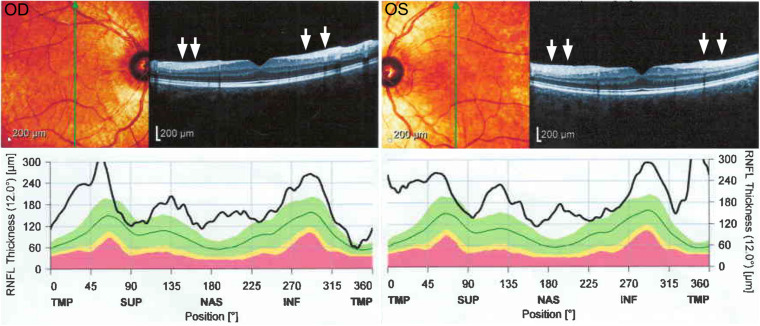
Optical coherence tomography of the right (OD) and left (OS) eye from patient 1 showing an increase in retinal nerve fiber layer thickness in all quadrants (white arrows) and uniformly increased values of peripapillary retinal nerve fiber layer thickness.

**FIGURE 2:**
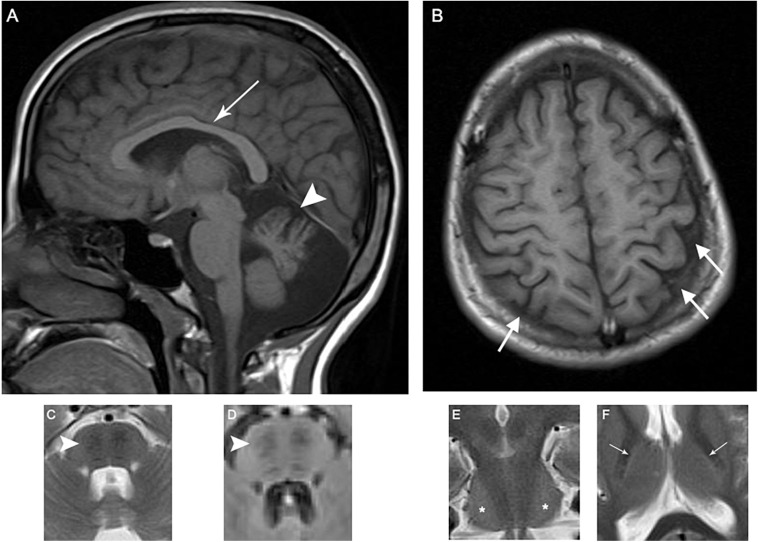
Patient 1 brain MRI revealing the characteristic ARSACS findings. MRI of the index patient reveals atrophy of the superior cerebellar vermis (arrowhead) and thinning of the posterior midbody of corpus callosum (white arrow) (**A**, T1); bilateral parietal atrophy, mostly prominent on the left side (white arrows) (**B**, T1); bilateral hypointense stripes in pons (arrowheads) (**C**, T2; **D**, FLAIR); bilateral hyperintensity of lateral pons (stars) on coronal images (**E**, T2); and a hyperintense rim around both thalami (white arrows) (**F**, T2).

Patient 1 is a 19-year-old female firstborn child of healthy, consanguineous parents. She had delayed developmental motor milestones and did not begin ambulating until the age of 3 years. Frequent falls, particularly during running, notably occurred during early childhood. At the age of 3 years, she developed ataxic gait and dysarthria that progressed over several years before plateauing; the number of falling episodes even decreased after the initiation of occupational therapy. At the time of examination, the patient was able to walk without any assistance. The patient performed normally at school and her cognitive function was normal according to Mini-Mental State Examination (score 30/30) (Ansari et al., 2010). She also complained of episodic muscle cramps, predominantly in the calf muscles. She neither had history of seizure, urinary or fecal incontinence, urinary urgency, nor audiologic, or visual problems. Neurologic examination at the age of 18 years demonstrated an ataxic gait with moderate dysarthria. Limb examination was significant for amyotrophy and *pes cavus* without any evidence of spasticity. Furthermore, bilateral sixth nerve palsies with saccadic pursuit and horizontal nystagmus were observed. In addition, mild rigidity after activation maneuvers (Supplementary Video 1), mirror movements (Supplementary Video 2), hypokinesia (Supplementary Video 3), and dysmetria were noticed in both upper extremities, predominantly on the left side. Although patellar reflexes were quite brisk, Achilles deep tendon reflexes, and deep tendon reflexes in the upper extremities were absent. Rapid elbow extension and supination of the forearm did not reveal any evidence of spasticity. Extensor plantar reflexes were present bilaterally (Babinski signs were present bilaterally). Nerve conduction studies and needle electromyography (EMG/NCS) were consistent with axonal sensorimotor polyneuropathy with some demyelinating features. Optical coherence tomography (OCT) revealed increased thickness of retinal nerve fiber layers (Figure 1). Echocardiography demonstrated myxomatous mitral valve leaflets without any evidence of hypertrophic cardiomyopathy. Pure tone audiometry and tympanometry were normal. Brain magnetic resonance imaging (MRI) depicted atrophy of the superior cerebellar vermis, thinning of the posterior mid-body of corpus callosum, bilateral parietal atrophy on T1-weighted images, bilateral hypointense stripes in the pons on T2/fluid-attenuated inversion recovery (FLAIR) sequences, bilateral hyperintensity of the lateral pons on coronal images on T2 sequences, and a hyperintense rim around both thalami on T2-weighted images (Figure 2). Laboratory investigations including serum vitamin B_12_, folate, vitamin E, thyroid function tests, anti-gliadin antibodies, lactate/pyruvate ratio, and blood and urine amino acid profiles were normal. After treating the patient with L-DOPA/benserazide 100/25 mg four times daily for 6 weeks, mild improvements in successive slowing and hesitation in finger tapping and hand movements were observed (Supplementary Videos 3–6). The patient also reported significant improvements in episodic muscle cramps during this period. Quality of life was assessed using the Persian version of the short form health survey (SF-36) before and 6 weeks after the treatment (Montazeri et al., 2005). Quality of life improved after the treatment from 54.85 to 57.42 with notable improvements in bodily pain and social functioning. She did not report any side effects during this 6-week period. The patient was also evaluated on two occasions after this period, and her motor function was assessed using Unified Parkinson’s Disease Rating Scale III (UPDRS-III) on and 48 h off medication, which showed a decrease in UPDRS-III scores from 17 to 12 on L-DOPA (Supplementary Videos 7–20). As a result, the patient was maintained on L-DOPA therapy.

Patient 2 is a 5-year-old boy who is the younger brother of patient 1. Like his sister, he suffered from developmental motor delay and started walking at the age of 2 years. He also had frequent falling episodes, especially while running. At the age of 3 years, he complained of calf muscle spasms. The patient also developed gait and truncal ataxia, which showed favorable response to occupational therapy; he can walk without using walking aids. His cognitive functions were normal, similar to his sister. Furthermore, no history of seizure or urinary or fecal incontinence was present. Neurologic examination at the age of 4 years revealed spastic ataxic gait without dysarthria. Mild weakness of the distal upper and lower extremities muscles was noted (4/5 MRC muscle scale). However, neither amyotrophy nor extremity deformities was present. Plantar reflexes were extensor bilaterally. Nevertheless, in contrast to patient 1, both Achilles deep tendon reflexes and patellar reflexes were brisk. EMG/NCS revealed evidence of demyelinating sensorimotor polyneuropathy. Brain MRI showed characteristic ARSACS neuroimaging findings, namely, superior cerebellar vermis atrophy, bilateral hypointense stripes in pons on T2 and FLAIR sequences, hyperintense lateral pontine T2/FLAIR changes, and a hyperintense rim around both thalami. An overview of the clinical and paraclinical findings of both patients is presented in Table 1.

**TABLE 1:** Clinical and paraclinical findings of the patients.

**Patient number**	**1**	**2**
Age, y/sex	19/Female	5/Male
Symptoms at onset	Developmental motor delay and frequent falls	Developmental motor delay and frequent falls
Spasticity	−	+
Babinski sign	+	+
Deep tendon reflex	Areflexia in both upper and lower limbs except for patellar reflexes which were quite brisk	Areflexia in upper limbs and brisk patellar and Achilles reflexes in lower limbs
Amyotrophy in upper and lower limbs	+	−
Muscle cramp	+	+
Gait ataxia	+	+
Dysarthria	+	−
Dysmetria	+	−
Nystagmus	+	−
Extrapyramidal signs	+	−
Mirror movements	+	−
Cognitive impairments	−	−
EMG/NCS	Axonal sensorimotor polyneuropathy with some demyelinating features	Demyelinating sensorimotor polyneuropathy
OCT	Increased thickness of retinal nerve fiber layers	NA
MRI findings	Atrophy of the superior cerebellar vermis, thinning of the posterior midbody of corpus callosum, bilateral parietal atrophy, bilateral hypointense stripes in pons on T2 and FLAIR sequences, bilateral hyperintensity of lateral pons on coronal images on T2 sequences, and a hyperintense rim around both thalami on T2-weighted images	Atrophy of the superior cerebellar vermis, bilateral hypointense stripes in pons on T2 and FLAIR sequences, bilateral hyperintensity of lateral pons on coronal images on T2 sequences, hyperintense rim around both thalami on T2-weighted images
Treatment	Occupational therapy + L-DOPA/benserazide	Occupational therapy

*ARSACS, autosomal recessive spastic ataxia of Charlevoix–Saguenay; F, female; M, male; EMG/NCS, electromyography/nerve conduction study; OCT, optical coherence tomography; NA, not available; MRI, magnetic resonance imaging; FLAIR, fluid-attenuated inversion recovery.*

### Molecular Findings

**FIGURE 3:**
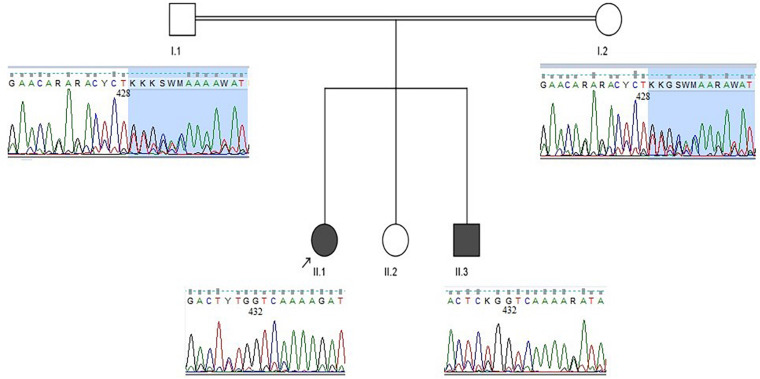
Family pedigree and sequence chromatograms. The two affected siblings (patient 1: II.1, patient 2: II.3) were homozygous for the identified *SACS* variant (c.429_430delTT), and both parents were heterozygous carriers.

Considering the mode of inheritance, clinical phenotypes, age at onset, and consanguineous marriage, a genetic disorder was suspected. Genomic DNA from the parents and both affected children was extracted from peripheral blood samples. Initially, we performed genetic testing for Friedreich ataxia, which failed to identify any pathological repeat expansions in the frataxin (FXN) gene in patient 1. Subsequently, whole-exome sequencing was carried out for patient 1, which revealed a novel homozygous deletion in the *SACS* gene (NM_014363.5:c.429_430delTT: p. Trp144ValfsTer39). Afterward, segregation analysis using Sanger sequencing was performed, which confirmed that her affected brother was homozygous for the identified mutation, and both parents were heterozygous carriers (Figure 3). No other pathogenic variants were identified in whole-exome sequencing study.

## Discussion

Autosomal recessive cerebellar ataxias constitute a heterogeneous group of disorders from both clinical and genetic perspectives (Beaudin et al., 2019; Synofzik et al., 2019). However, they each share the neuropathological hallmark of progressive damage to the cerebellum and its associated tracts (Anheim et al., 2012). Pathogenic mutations in the *SACS* gene are known to cause ARSACS and have been identified in patients from all over the world, even including remote regions in India (Kuchay et al., 2019; Xiromerisiou et al., 2019). We investigated the clinical and molecular findings in two Iranian individuals affected with this disorder and identified a novel homozygous mutation in *SACS* gene in both of them, expanding not only the mutational spectrum and geographical distribution of this disorder but also its clinical features.

Historically reported clinical presentations of ARSACS including ataxia, corticospinal tract involvement, and axonal neuropathy were present in the patients described here (Pilliod et al., 2015). Likewise, thickening of retinal nerve fiber layers, which has been suggested as a diagnostic marker in ARSACS, was detected in patient 1 described here (Parkinson et al., 2018; Kuchay et al., 2019). Characteristic neuroimaging features of ARSACS were present in both affected individuals. In particular, T2 hyperintense rims around both thalami (“thalamic rims”) were seen in both cases, providing further support for the utility of this neuroimaging diagnostic marker for ARSACS (Kuchay et al., 2019).

Patient 1 had rigidity, hypokinesia/bradykinesia, and mirror movements. To the best of our knowledge, these findings have not been reported previously in other patients with ARSACS. Brain lesions in a wide range of locations functionally connected to a common network of regions encompassing brainstem, thalamus, cingulate cortex, basal ganglia, and cerebellum have been shown to lead to parkinsonism (Joutsa et al., 2018), with the latter location potentially explaining our observations in this patient. This movement disorder cannot be explained well by an alternative explanation of pyramidal slowing (i.e., slowing of movements seen in pyramidal tract disorders) as both fatiguing and in particular successive decrement in the amplitude of repetitive movements were present (Edwards et al., 2016). Furthermore, partial improvements observed after L-DOPA intake are suggestive of non-pyramidal and hypokinetic/bradykinetic signs. The absence of these findings in the other sibling might be due to his younger age and therefore being in the earlier stages of the disease course.

Moreover, altered interhemispheric inhibition due to corpus callosal atrophy and consequently increased cortical motor outflow might explain the occurrence of mirror movements in our patient. In comparison with Quebec patients, those with ARSACS originating from other parts of the world often develop more atypical clinical presentations (Takiyama, 2007).

Both affected individuals had episodic muscle cramps, which was the most bothersome symptom in patient 2. In a study of 43 patients with ARSACS, muscle spasms were the most frequent finding reported outside the classic triad, being present in up to 82% of older (≥40 years) participants (Briand et al., 2019). Considering the growing interest in the development of targeted molecular therapies for this group of disorders, therapeutic trials will be available soon (Synofzik et al., 2019). Hence, it is of cardinal importance to include this agonizing symptom as a decisive measure of efficacy of any intervention in potential clinical trials in the future. Although our study is very limited, dramatic improvements in episodic muscle cramps in patient 1 following treatment with L-DOPA/benserazide are a notable finding.

Although cognitive dysfunction and intellectual disability are reported to be present in more than half of the patients, both patients described here were cognitively normal (Pilliod et al., 2015). Neuroimaging studies in both patients revealed hypointense stripes in the pons and atrophy of the superior cerebellar vermis, which was similar to previous reports of patients with cognitive and neuropsychiatric presentations (Mignarri et al., 2014). Further functional neuroimaging studies in ARSACS patients with varying cognitive dysfunction might shed further light on the role of the cerebellum in cognitive and affective disorders.

The major limitation of our study was the limited number of patients and also lack of advanced neuroimaging studies (e.g., DaTscan) to further characterize the underlying pathology. Nevertheless, our findings expand the genetic and phenotypic spectrum of ARSACS. Although specific underlying aberrations in neuronal circuitries remain elusive, this study provides fresh impetus for further investigations on the role of cerebellum in movement disorders.

## Data Availability Statement

The original contributions presented in the study are included in the article/Supplementary Material, further inquiries can be directed to the corresponding author/s.

## Ethics Statement

The studies involving human participants were reviewed and approved by Persian BayanGene Research and Training Center Ethics Committee. Written informed consent to participate in this study was provided by the participants’ legal guardian/next of kin. Written informed consent was obtained from the individual(s), and minor(s)’ legal guardian/next of kin, for the publication of any potentially identifiable images or data included in this article.

## Author Contributions

MF conceived and designed the study, collected, assembled, and interpreted NGS data. PH, VO, and SI clinically evaluated the patients. PH drafted the manuscript. PH and ZT performed the genetic studies. MS, VO, MF, and MN revised the manuscript. All authors contributed to the article and approved the submitted version.

## Conflict of Interest

The authors declare that the research was conducted in the absence of any commercial or financial relationships that could be construed as a potential conflict of interest.

## Supplementary Materials

Supplementary Video 1 Rigidity after activation maneuver in patient 1.

Supplementary Video 2 Mirror movements in patient 1.

Supplementary Video 3 Hand movements in patient 1 before treatment.

Supplementary Video 4 Hand movements in patient 1 six weeks after treatment with L-dopa/benserazide.

Supplementary Video 5 Finger tapping in patient 1 before treatment.

Supplementary Video 6 Finger tapping in patient 1 six weeks after treatment with L-dopa/benserazide.

Supplementary Video 7 Finger tapping in patient 1 ON medication.

Supplementary Video 8 Finger tapping in patient 1 OFF medication.

Supplementary Video 9 Hand movements in patient 1 ON medication.

Supplementary Video 10 Hand movements in patient 1 OFF medication.

Supplementary Video 11 Supination pronation of hand in patient 1 ON medication.

Supplementary Video 12 Supination pronation of hand in patient 1 OFF medication.

Supplementary Video 13 Left toe tapping in patient 1 ON medication.

Supplementary Video 14 Left toe tapping in patient 1 OFF medication.

Supplementary Video 15 Right toe tapping in patient 1 ON medication.

Supplementary Video 16 Right toe tapping in patient 1 OFF medication.

Supplementary Video 17 Left leg agility in patient 1 ON Medication.

Supplementary Video 18 Left leg agility in patient 1 OFF Medication.

Supplementary Video 19 Right leg agility in patient 1 ON Medication.

Supplementary Video 20 Right leg agility in patient 1 OFF Medication.
